# Tubulocystic renal cell carcinoma in the left kidney: a case report

**DOI:** 10.1186/1752-1947-8-265

**Published:** 2014-07-31

**Authors:** Yusuke Ishibashi, Takuya Koie, Naoki Fujita, Tendo Satoh, Jotaro Mikami, Shingo Hatakeyama, Chikara Ohyama, Yuki Tobisawa, Tohru Yoneyama

**Affiliations:** 1 Department of Urology, Hirosaki University Graduate School of Medicine, 5 Zaifucho, Hirosaki 036-8562, Japan

**Keywords:** Collecting duct carcinoma, Tubulocystic renal cell carcinoma

## Abstract

**Introduction:**

Tubulocystic renal carcinoma is a rare tumor and has been recently recognized as a neoplastic entity. We report a case of tubulocystic renal carcinoma in the left kidney and present a review of relevant literature.

**Case presentation:**

A 35-year-old Japanese woman visited our hospital with the chief complaint of left-sided back pain. Computed tomography revealed a hemorrhagic cyst (size, 7×8cm) in the upper pole of her left kidney. Approximately 3 years after the initial diagnosis, she complained of left-sided back pain again. Magnetic resonance imaging revealed an enlarged left renal cyst (size, 10×12cm) with a slightly enhanced cystic wall. The tumor was clinically diagnosed as a renal cell carcinoma in the cT2N0M0 stage, which arose from the cyst wall; therefore, left nephrectomy was performed. On histological examination, the tumor showed circumscribed proliferation with cystically dilated tubules. The tubules and cysts were lined by a single layer of flat, hobnail, and cuboidal cells. Immunohistochemical analysis revealed that the tumor cells were strongly positive for E-cadherin and P504S.

**Conclusions:**

Examination of more cases of tubulocystic renal carcinoma is required to better understand the biology of this tumor and to ascertain its prognosis.

## Introduction

Tubulocystic renal carcinoma (TCRC) is a rare tumor, and has been recently recognized as a neoplastic entity. TCRC was not formally described in the World Health Organization 2004 classification or the guidelines given in the 2004 Armed Forces Institute of Pathology fascicle [1,2]; however, Amin *et al*. were the first to report about TCRC on the basis of its characteristic morphology [3]. Here we report a case of TCRC in the left kidney and present a review of the relevant literature.

## Case presentation

**Figure 1:**
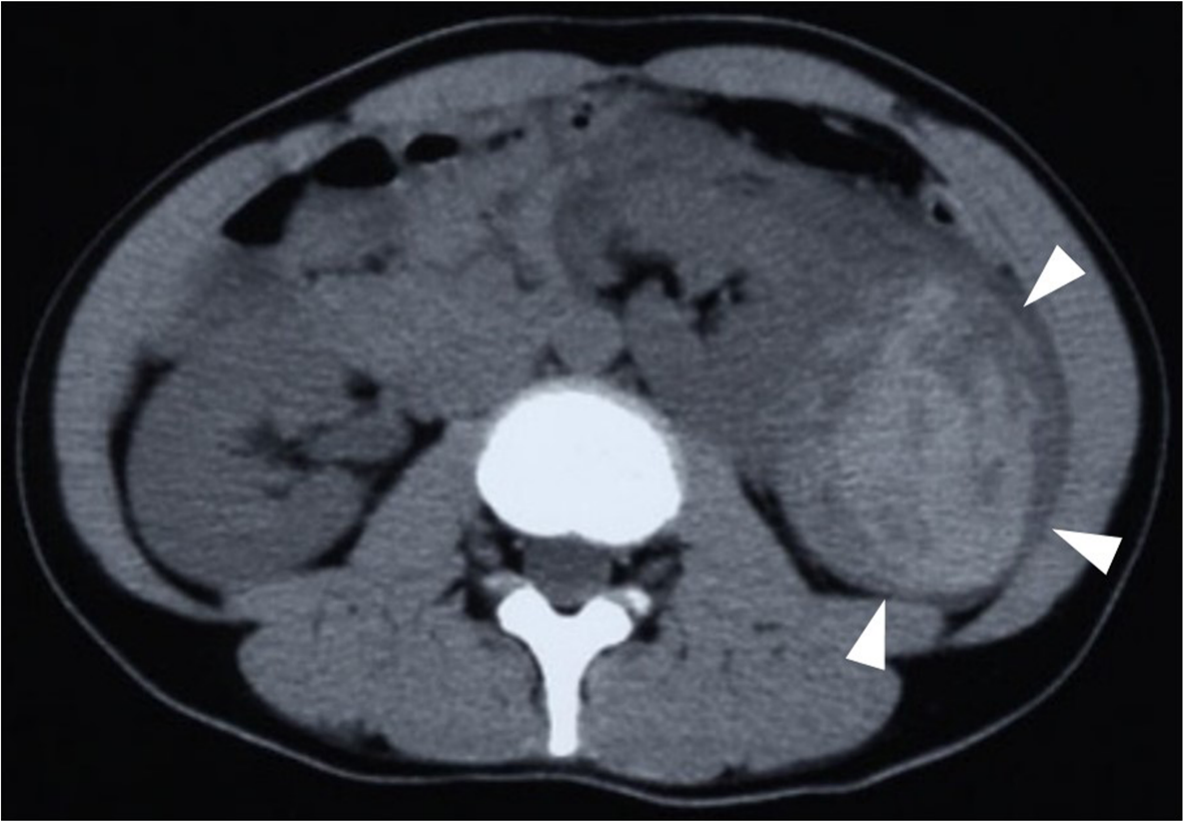
**Enhanced abdominal computed tomography.** An abdominal computed tomography image showing a hemorrhagic cyst in the upper pole of the left kidney (arrowheads).

**Figure 2:**
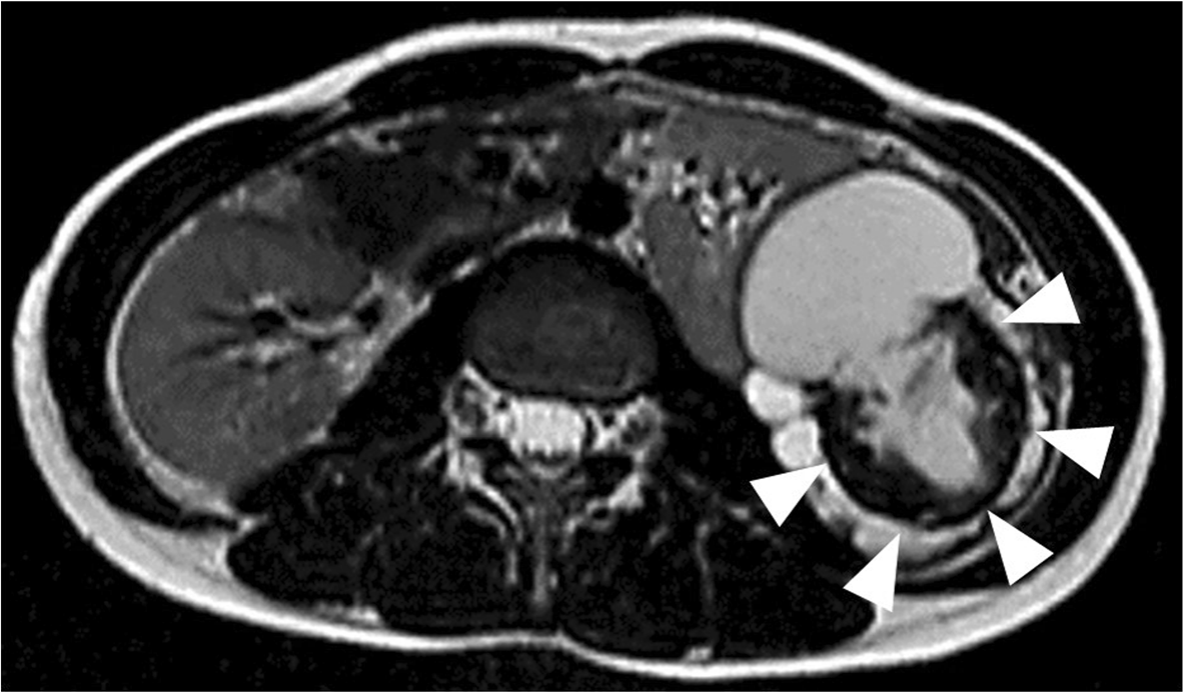
**Abdominal magnetic resonance imaging.** A T2-weighted magnetic resonance image showing a high-intensity tumor (arrowheads).

**Figure 3:**
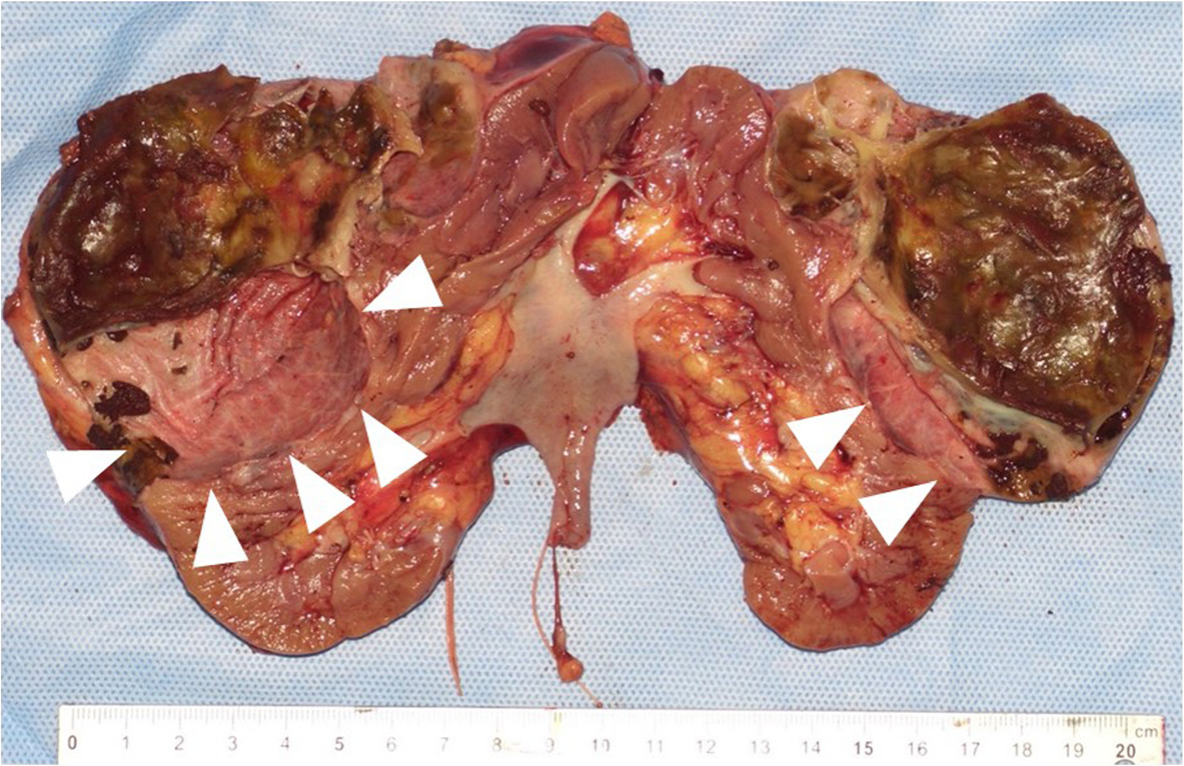
**Macroscopic findings.** A white/gray spongy cut surface measuring 10×12cm in size can be seen in the upper pole of the resected kidney (arrowheads).

**Figure 4:**
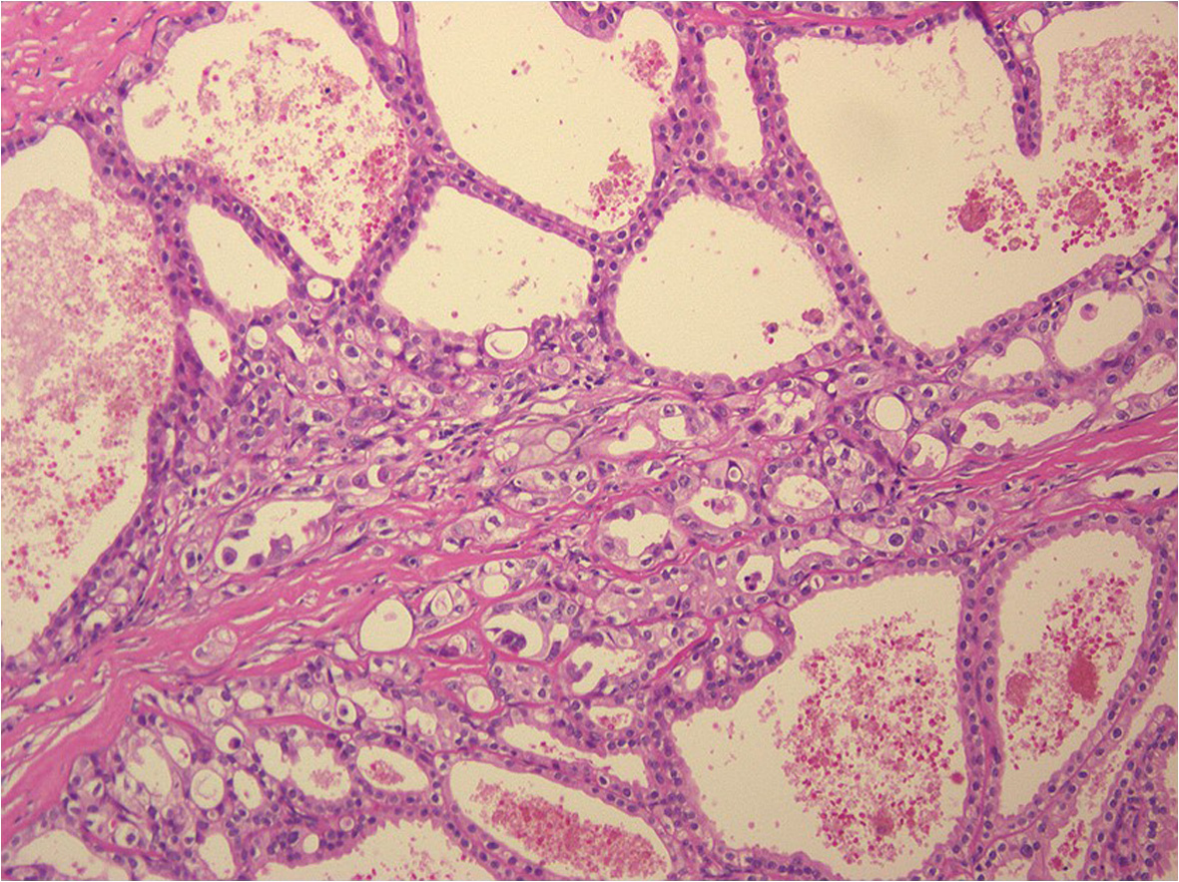
**Hematoxylin-eosin staining.** Hematoxylin-eosin-stained section showing cystically dilated kidney tubules lined with cuboidal and flat cells (magnification, 100×).

**Figure 5:**
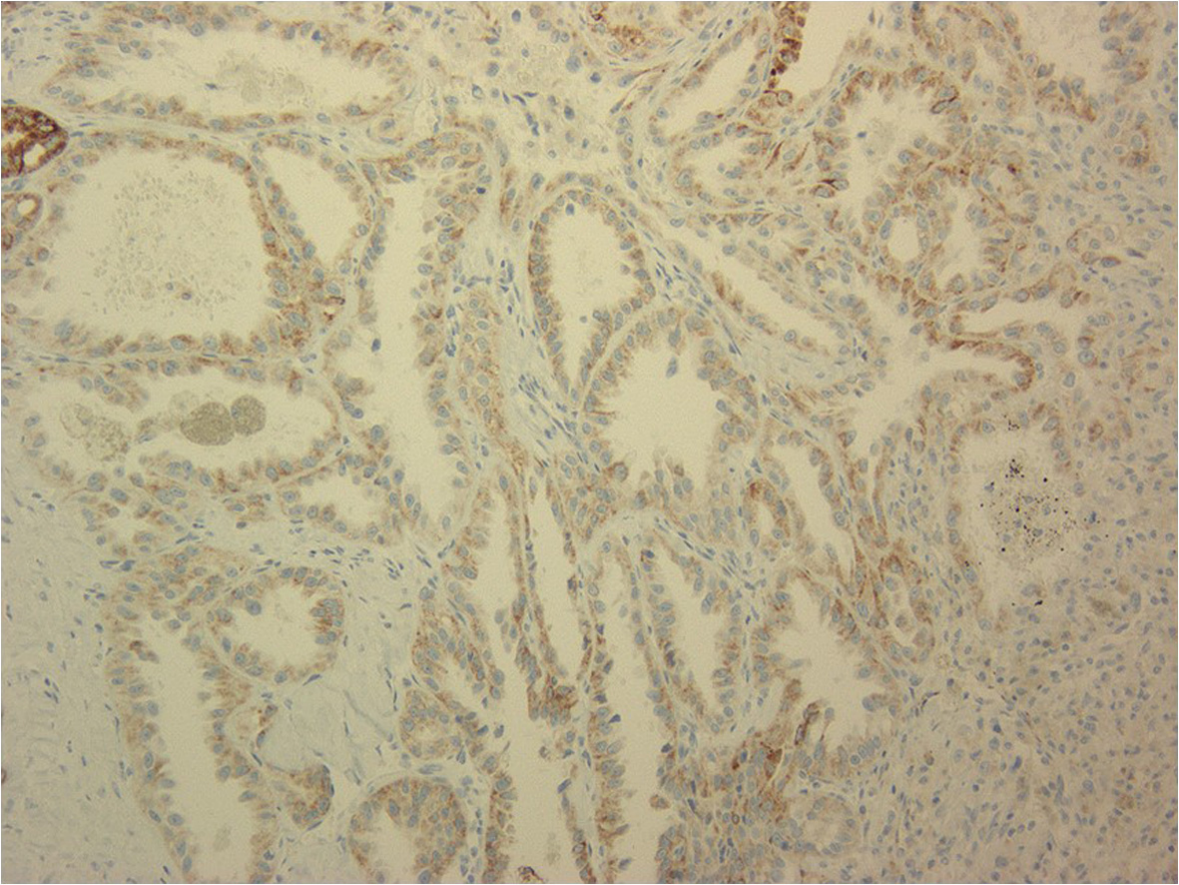
**Immunohistochemical findings for P504S.** The tumor cells were strongly positive for P504S (magnification, 200×).

**Figure 6:**
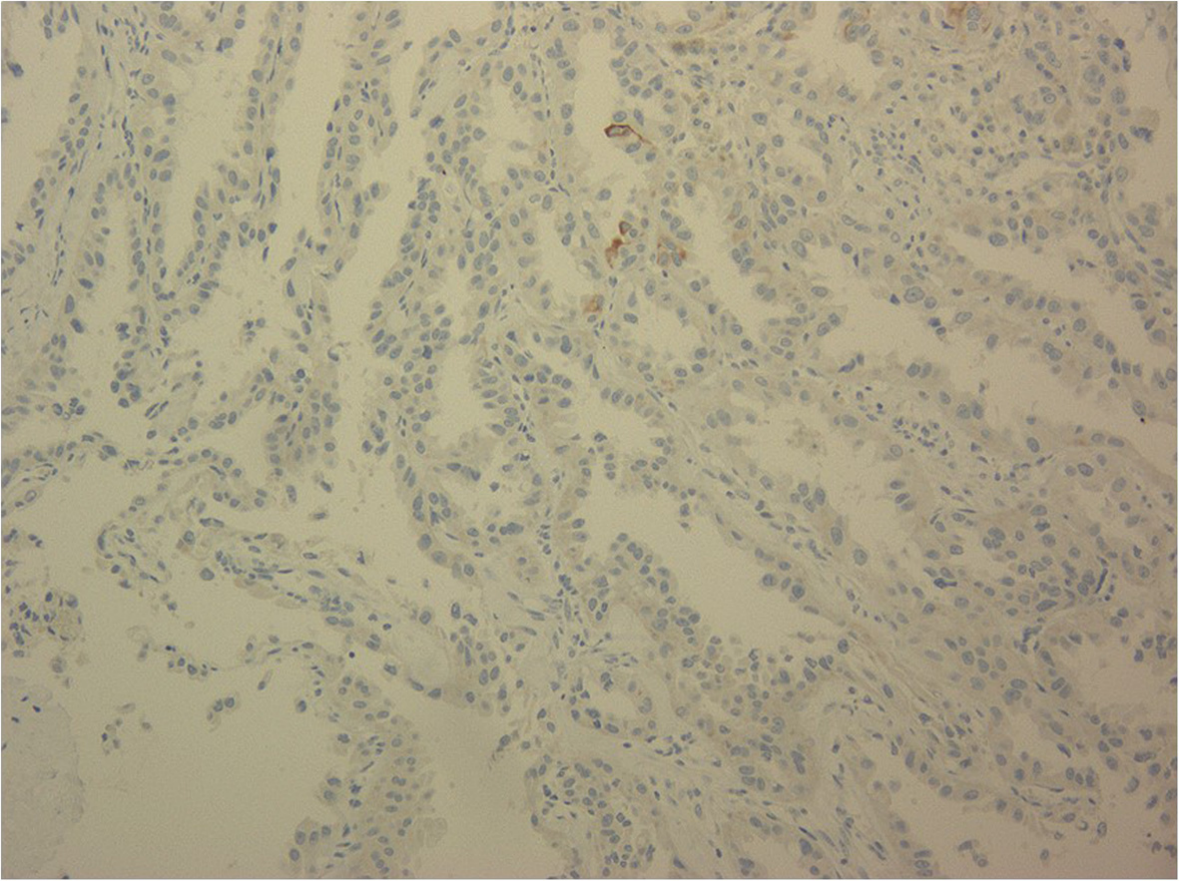
**Immunohistochemical findings for cytokeratin 34β12.** The tumor cells were negative for cytokeratin 34β12 (magnification, 200×).

A 35-year-old Japanese woman visited our hospital with the chief complaint of left-sided back pain. Computed tomography revealed a hemorrhagic cyst (size, 7×8cm) in the upper pole of her left kidney (Figure 1). She was relatively young, but she rejected further examination or surgical intervention. She was followed-up every 6 months by using magnetic resonance imaging. During the follow-up period, she did not have any complaints, including back pain. Approximately 3 years after the initial diagnosis, she complained of left-sided back pain again. Magnetic resonance imaging revealed an enlarged left renal cyst (size, 10×12cm), with a slightly enhanced cystic wall (Figure 2). The radiological diagnosis was used to classify the cyst as a Bosniak type III cyst. The tumor was clinically diagnosed as a left renal cell carcinoma (RCC) that arose from the cyst wall, and was classified as a cT2N0M0 tumor, according to the tumor-node-metastasis system. Left nephrectomy was performed. Macroscopic analysis of the upper pole of the resected kidney revealed the presence of clustered smooth-walled cysts with few solid areas (Figure 3). On histological examination, the tumor showed circumscribed proliferation with cystically dilated tubules. The tubules and cysts were lined by a single layer of flat, hobnail, and cuboidal cells (Figure 4). Papillary structures were observed within the cysts. Immunohistochemical analysis revealed that the tumor cells were strongly positive for E-cadherin and P504S (Figure 5). The tumor cells did not stain positive for WT1, cytokeratin (CK) 34β12, transcription factor E3 (TFE3), and CD10 (Figure 6). On the basis of these findings, she was diagnosed with TCRC in her left kidney.

Subsequently, she has been followed-up for 5 years without any evidence of local recurrence or distant metastasis.

## Discussion

Masson originally described TCRC with Bellinian epithelioma as tumors of the collecting ducts of Bellini [4]. Therefore, an evolving concept of collecting duct carcinomas was proposed, and low-grade collecting duct carcinoma at the beginning of the spectrum corresponds to the current TCRC [5]. However, by using molecular clustering, TCRC was found to be more closely related to papillary RCC than to any other renal neoplasm such as collecting duct carcinoma [6]. Supportive findings include the expression of proximal convoluted tubule markers (CD10 and alpha-methylacyl-CoA racemase; AMACR), distal nephron proteins (parvalbumin, high-molecular-weight CK, and CD19) [3,7], and the detection of intercalated cells and cells similar to those in the proximal tubule and by using electron microscopy [3,8,9]. In addition, Alexiev and Drachenberg [10] reported that all TCRCs were strongly positive for the proximal convoluted tubule markers CD10, vimentin, and AMACR, but they were only focally positive for BerEP4 or focally negative for CK34β12, both of which are distal nephron proteins. By using fluorescence *in situ* hybridization and array-based comparative genomic hybridization, Yang *et al*. [6] and Zhou *et al*. [8] have shown gains in chromosome 17, as is seen in patients with papillary RCCs; thus, they have proposed that these two entities are closely related.

TCRC is a tumor that occurs in adults with a wide age range, but most patients present with TCRC in the fifth or sixth decade [3,6]. There is a strong predominance of TCRC among men (male/female ratio of 7:1), and approximately 60% of reported TCRC cases are observed in the left kidney [3,6]. Patients are often asymptomatic, although they may present with abdominal pain, abdominal distention, and hematuria [10]. TCRCs are usually small in size at presentation, with nearly 40% of the reported cases involving tumors that measure ≤2cm [6,7]. On macroscopic examination, TCRCs are circumscribed and unencapsulated, and they demonstrate a white or gray spongy cut surface that is often compared with “bubble wrap” [6].

The differential diagnosis of TCRC is interesting, and it mostly includes other tumors with a multiloculated gross appearance such as multilocular cystic RCC, cystic nephroma, mixed epithelial and stromal tumors, cystic oncocytoma, and Xp11.2 RCC [11,12]. Multilocular cystic RCC shows an innate multicystic architecture with variably sized cystic spaces lined by flattened to cuboidal clear cells similar to those seen in clear RCC [13,14]. Cystic nephroma shows the presence of multilocular cysts lined by flattened to attenuated epithelial cells; hobnailing may occasionally be noted and is infrequently quite prominent [15]. Mixed epithelial and stromal tumors have solid areas and broad septa, in contrast to the thin fibrous septa of TCRC [10]. Cystic oncocytomas usually show, at least focally, a solid nest of oncocytic cells along with loose myxoid stroma [10]. Xp11.2 RCC with a tubulocystic pattern is rare. Immunohistochemical analysis for TFE3 and cathepsin K is helpful in the differential diagnosis of Xp11.2 RCC [16]. The architecture is predominantly cystic with large cysts and hyalinized stroma, fibrotic, or may infrequently have an ovarian stroma-like character [13,17]. TCRC shows the presence of tightly packed tubules and cysts measuring up to a few millimeters in diameter, which are separated by bland fibrous stroma [11]. The lining cells are cuboidal to columnar and may have an attenuated appearance [11]. Hobnail cells are commonly observed. Both TCRCs and cystic nephromas have cysts that are lined by hobnail cells, but the cells have a low nuclear grade in cystic nephroma [11]. In addition, the stroma is paucicellular and fibrotic in TCRCs [11].

The biological behavior of TCRCs has not been fully established. According to previous reports, a total of six patients have developed metastatic diseases [3,6,11]. Srigley and Delahunt [11] reported that two patients (9.1%) developed distant metastases to the bone or liver. Interestingly, the two metastatic tumors displayed areas of clear cells. Although the majority of tumors behave in an indolent fashion, one needs to be cautious in rendering a prognosis.

## Conclusions

TCRCs are a distinctive group of kidney tumors that are predominant in men, and have noteworthy macroscopic (“bubble wrap” appearance) and microscopic (cysts lined with hobnail cells and separated by a thin fibrotic stroma) characteristics. The examination of more cases is required to better understand the biology of this tumor, ascertain its prognosis, and choose the appropriate treatment.

## Consent

Written informed consent was obtained from the patient for publication of this case report and the accompanying images. A copy of the written consent is available for review by the Editor-in-Chief of this journal.

## Abbreviations

AMACR: Alpha-methylacyl-CoA racemase; CK: Cytokeratin; RCC: Renal cell carcinoma; TCRC: Tubulocystic renal carcinoma; TFE3: Transcription factor E3.

## Competing interests

The authors declare that they have no competing interests.

## Authors’ contributions

YT drafted the manuscript. TK was involved in the drafting of the manuscript. NF, TS, and JM performed the clinical follow-up. YI and SH performed the operation. YT and TY analyzed the pathological specimens. TK and CO were responsible for the conception and design of this study, interpretation of the data, and critical revision of the manuscript. All authors have read and approved the final manuscript.

## Authors’ information

YI Clinical Fellow, TK Associate Professor, NF Clinical Fellow, TS Clinical Fellow, JM Clinical Fellow, SH Lecturer, CO Professor and Chairman, YT Assistant Professor, TY Assistant Professor.
